# Effect of Palladium Electrode Patterns on Hydrogen Response Characteristics from a Sensor Based on Ta_2_O_5_ Film on SiC at High Temperatures

**DOI:** 10.3390/s19245478

**Published:** 2019-12-12

**Authors:** Kyeong-Keun Choi, Seongjeen Kim

**Affiliations:** 1 National Institute for Nanomaterials Technology (NINT), Pohang University of Science and Technology (POSTECH), Pohang 37666, Korea; choikk@postech.ac.kr; 2 Department of Electronic Engineering, Kyungnam University, Changwon 51767, Korea

**Keywords:** hydrogen sensor, silicon carbide, palladium electrode, pattern type, high temperature, tantalum oxide

## Abstract

Our study aims to fabricate a hydrogen sensor based on thermal stability analysis of Ta_2_O_5_ film, and to determine the effect of Pd electrodes on the hydrogen sensor at high temperatures. First, in order to ensure high-temperature stability of silicon carbide (SiC)-based hydrogen sensors, the thermal stability of Ta_2_O_5_ dielectric thin film at temperatures above 900 °C was studied. The sensor structure consisted of a metal-insulator-semiconductor (MIS) and a tantalum oxide (Ta_2_O_5_) dielectric film was formed by rapid thermal oxidation (RTO). The Ta_2_O_5_ film was assessed through SEM, TEM, SIMS, and dielectric breakdown strength to observe thermal stability. Secondly, hydrogen sensors using a SiC substrate were fabricated, with the process considering thermal stability. The response characteristics for hydrogen were evaluated using three types of sensors with different Pd electrode patterns. The patterns of the Pd electrode were designed as squares or grid shapes, and were characterized by 100%, 75%, and 50% area ratios of Pd electrodes covering the Ta_2_O_5_ layer. The results showed that the sensor with a 100% area ratio of the Pd electrode had better sensitivity and linear response characteristics compared to sensors with a 50% area ratio of the Pd electrode.

## 1. Introduction

The amount of hydrogen gas in the atmosphere is very small; it makes up about 0.5 ppm. At standard temperature and pressure, hydrogen is a colorless, odorless, tasteless, and nontoxic gas. However, hydrogen gas is highly explosive if it reaches a concentration of more than 4% in the atmosphere and is a major cause of metal corrosion. Thus, devices and facilities that handle hydrogen gas need to be equipped with reliable sensors to detect hydrogen leaks. In particular, in the case of hydrogen vehicles that use compressed hydrogen stored at high pressure, installation of hydrogen sensors is essential.

Semiconductor gas sensors have generally been manufactured using silicon substrates with stable process technology [1], but sensors that use silicon as a substrate have a serious disadvantage in that they cannot operate at temperatures above 250 °C due to their relatively small band gap energy (E_G_ = 1.12 eV). However, there are numerous high-temperature facilities that achieve temperatures of over 250 °C, for example, furnaces in steel mills, chemical reactors, and automobile and aviation engines. Therefore, sensors that can operate even in high-temperature environments are needed. The development of sensors that can operate at high temperatures is essential in terms of expanding their application and the development of different materials, as well as improving the configuration of the detection circuit system. The electrical properties of semiconductors are sensitive to temperature. Silicon carbide (SiC), gallium nitride (GaN), and gallium phosphide (GaP), which have large band gap energy, are relatively suitable for high-temperature applications. Meanwhile, studies using ceramic substrates including Al_2_O_3_ as an insulator for high-temperature gas sensors have been conducted [2,3,4].

To date, various materials and production methods have been used to produce hydrogen sensors able to withstand high temperatures [5,6,7,8,9,10]. The first study investigating a high-temperature hydrogen sensor using SiC as a substrate was performed in 1992 [11]. The sensor comprised a simple Schottky diode structure, consisting of only one metal electrode on the SiC substrate. However, this structure was not stable after long-term high-temperature operations due to its poor interface properties. Structures of semiconductor sensors may roughly be divided into two types: the Schottky barrier and the metal-insulator-semiconductor (MIS) structure. Gas sensors characterized by a MIS have advantages such as a simple structure, reduction of the interfacial diffusion, and thermal transfer blocking by the oxide film between the metal electrode and the semiconductor substrate. Therefore, they are suitable for higher temperature use than the Schottky contact structure. In addition, the capacitive-type sensors [12,13] in the MIS structure are less sensitive to temperature changes than the resistive-type [14,15], therefore, they are more suitable for environments with high-temperature variations because they require almost no temperature compensation.

We have reported a study evaluating a hydrogen sensor using a tantalum oxide (Ta_2_O_5_) layer on the SiC substrate, which is suitable for high temperatures [12,16]. Ta_2_O_5_ possesses many desirable features, including good chemical resistance and low light absorption, as well as high permeability for hydrogen gas with a high dielectric constant of about 25 and barrier heights appropriate to the SiC substrate [17]. In the study, a Ta_2_O_5_ film is formed by rapid thermal oxidation (RTO), which has the advantage of reducing defects in the Ta_2_O_5_ layer, as well as dopant penetration and interface reactions at the interface used. However, it has also been reported that crystallization of the Ta_2_O_5_ film affects electrical properties. The Ta_2_O_5_ layer shows a drastic increase in leakage current in the crystalline state [18,19]. We compared the interface of the Ta_2_O_5_/substrate formed by RTO on silicon (Si) and SiC substrates using transmission electron microscopy (TEM) and secondary ion mass spectrometry (SIMS) at RTO temperatures of 900 to 1000 °C, and then measured the I–V curves to determine its dielectric breakdown strength.

The choice of the metal electrode and of the oxide film is important in hydrogen sensors. Palladium (Pd) has the ability to absorb large volumetric quantities of hydrogen at room temperature and atmospheric pressure, and subsequently contributes to the formation of palladium hydride (PdH*_x_*) within the dielectric thin film [20,21,22,23]. In this study, in order to assess the effects of the Pd electrode on the response characteristics of hydrogen sensors, three types of sensors with different Pd electrode patterns were applied to the MIS structure. The capacitive response characteristics of the sensors were estimated for hydrogen concentrations ranging up to 2000 ppm. As a result, it was confirmed that the Pd electrode exerted a significant effect on the hydrogen response.

## 2. Experiments

**Figure 1:**
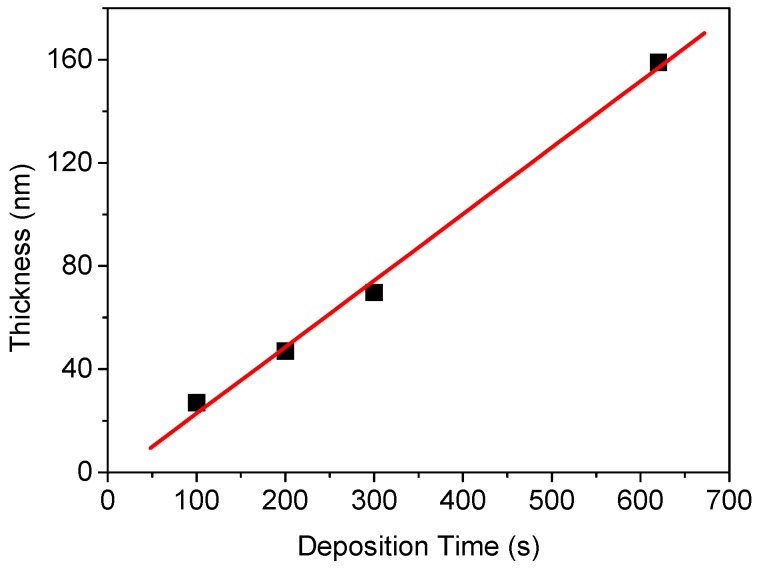
Ta film thickness as a function of deposition time at power of 600 W, pressure of 2 mTorr, and Ar flow rate of 20 sccm.

In this study, n-type 4H–SiC wafers were used as substrates to produce hydrogen sensors for high-temperature application. The SiC substrate size was 4 inch (or 1.2 × 1.2 cm^2^ in size). After pre-cleaning, tantalum (Ta) films were deposited on the SiC substrate at power of 600 W, pressure of 2 mTorr, and argon (Ar) flow rate of 20 sccm. Then, the Ta_2_O_5_ layer was formed using an RTO process for two minutes at a temperature range of 900 to 1000 °C. Figure 1 shows the relationship between deposition time and formation thickness of tantalum. Applying this process, tantalum with a thickness of ~50 nm was formed at a flow rate of 200 s.

**Figure 2:**
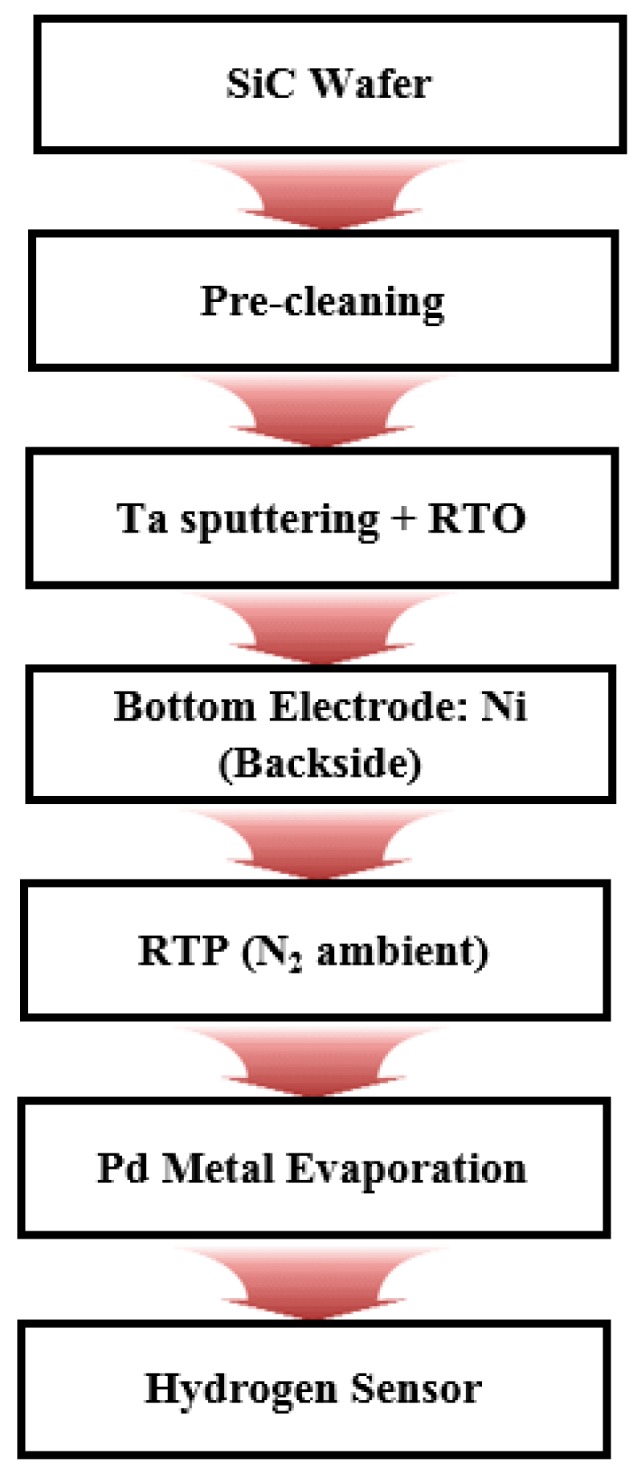
Schematic flowchart of experimental procedure.

A nickel (Ni) film was deposited on the backside of the wafer to form electrodes, and the annealing process was conducted for one minute at 950 °C for ohmic contact, after which Pd was deposited for 2 min at 300 W power with shadow masks of three different patterns in order to form the front Pd electrodes. The SiC substrates (hydrogen sensors) were attached to the quartz plates within a 3 × 3 cm^2^ area and were finished with wire bonding. Figure 2 shows the overall process flowchart for fabricating the hydrogen sensors with the Pd (top)/Ta_2_O_5_/SiC/nickel silicide (NiSi*_x_*) structure.

**Figure 3:**
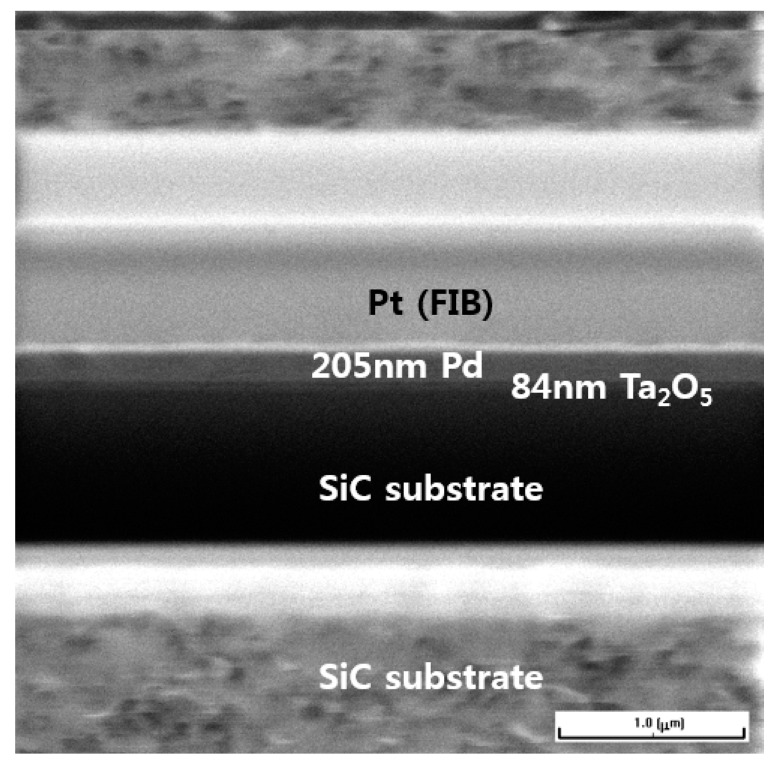
A focused ion beam (FIB)-scanning electron microscope (SEM) image of the cross-section of the sample.

The sensors are made of a MIS structure. Pd film with a thickness of 200 nm was deposited over the Ta_2_O_5_ layer. Figure 3 shows a focused ion beam (FIB)-SEM image of the cross-section of the sensor. Initially, the thickness of the tantalum film was 50 nm, but after the RTO process the thickness of the Ta_2_O_5_ layer increased to approximately 80 nm. The thickness of NiSi*_x_* used as the bottom-side electrode was determined to be approximately 100 nm. In a previous study [24], it was reported that the thermally stable Ni–Si silicide layers had excellent ohmic properties and no hysteresis behavior was observed on 4H–SiC substrates after rapid thermal annealing (RTA) at temperatures of 925 to 975 °C. Platinum (Pt) in the FIB-SEM image indicates a layer formed during the sample preparation by the FIB to protect the Pd layer from damage.

The electrical properties of sensors were determined using a semiconductor device analyzer and an inductance-capacitance-resistance (LCR) meter. The sensors were measured in a chamber capable of temperature adjustments. The chamber was composed of a quartz tube 75 mm in diameter with a cooling system. The concentration of hydrogen gas was adjusted to 4 stages of 0, 500, 1000, and 2000 ppm, using the mass flow controller (MFC). The sensors were flushed with clean nitrogen gas before exposure to hydrogen gas. Our measurements were basically conducted in a dry atmosphere, where the relative humidity was always less than 20%.

## 3. Results and Discussion

### 3.1. Evaluation of Ta_2_O_5_ Properties in the MIS Structure

A Ta_2_O_5_ film formed by RTO was used as the dielectric layer. Ta_2_O_5_ films produced by RTO have been characterized for their advantages of reducing cracks, pinholes, and pores in the Ta_2_O_5_ layer, and for advantages of the dopant diffusion penetration at the interface of Ta_2_O_5_ and substrate [18]. However, it has also been reported that the crystallization of the Ta_2_O_5_ film affects electrical properties. The Ta_2_O_5_ layer shows excellent electrical characteristics in the amorphous state. However, it is crystallized at temperatures above 700 °C, resulting in a drastic increase in the leakage current [19]. This means that an analysis of the optimized post-process to reduce the leakage current in the MIS structure and the thermal stability of hydrogen sensors in high-temperature applications is needed.

**Figure 4:**
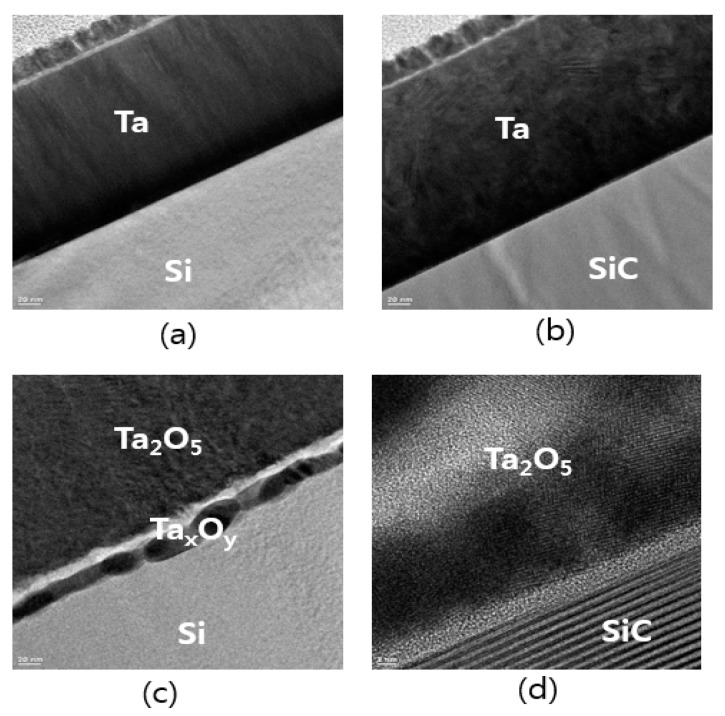
(**a**,**b**) Cross-sectional TEM images of deposited Ta layers and (**c**,**d**) tantalum oxide (Ta_2_O_5_) layers; (**a**,**c**) on Si substrate and (**b**,**d**) on silicon carbide (SiC) substrate.

We compared the interface of Ta_2_O_5_/substrates formed by RTO deposited on Si and SiC substrates using TEM at an RTO temperature from 900 °C to 1000 °C in order to assess the uniform distribution of components at the interface. Figure 4 shows the cross-sectional TEM images of as-deposited Ta layers (Figure 4a,b) and Ta_2_O_5_ layers after RTO for 2 min in 5 standard liters per minute (SLPM) ambient oxygen (Figure 4c,d). TEM images show deposition on Si (Figure 4a,c) and on SiC substrates (Figure 4b,d). Following RTO, the interface of the Ta_2_O_5_ layer/substrate formed on SiC substrates was more uniform than that of the interface of the Ta_2_O_5_/substrate.

**Figure 5:**
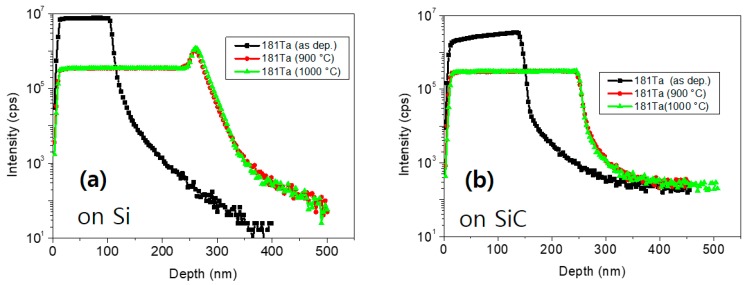
Secondary ion mass spectrometry (SIMS) depth profiles of Ta atoms according to rapid thermal oxidation (RTO) temperature (**a**) on Si substrates and (**b**) on SiC substrates.

This result was confirmed by SIMS measurement. Figure 5 shows SIMS depth profiles of Ta atoms deposited on Si and SiC substrates, respectively, before and after RTO above 900 °C for 2 min. The Ta atoms accumulated at the interface of the Ta_2_O_5_/Si substrate after RTO above 900 °C, whereas there was no such accumulation at the interface of the Ta_2_O_5_/SiC substrate (Figure 5a). Therefore, it was confirmed that the Ta_2_O_5_ layer on the SiC substrate is a more stable substrate than that on the Si substrate at high temperatures over 900 °C.

**Figure 6:**
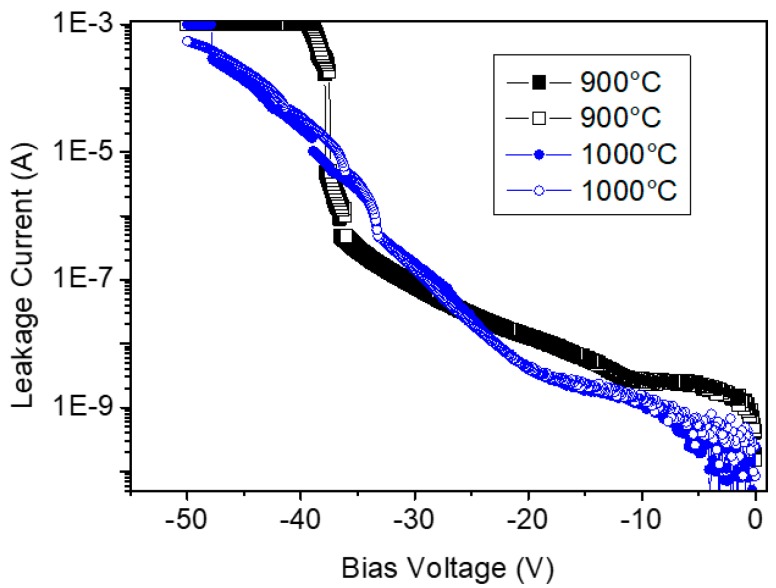
Properties of the I–V in a metal–insulator–semiconductor (MIS) structure with ~250 nm thick tantalum oxide layers formed at 900 °C (black line) and 1000 °C (blue line).

Figure 6 shows the current–voltage (I–V) properties for the samples in the MIS structure consisting of Pd electrode and Ta_2_O_5_ dielectric layer on the SiC substrate. The voltage was applied with a step of 0.1 to −50 V. The direction of the applied voltage was based on the Pd electrode, and the thermal treatment temperature of the Ta_2_O_5_ layer ranged from 900 °C to 1000 °C before Pd film deposition. The breakdown field was shown to be ~1.5 MV/cm. In general, the operating voltage of the sensor was <10 V. The leakage current was very low in the low electrical field even though it was shown to increase over −20 V.

### 3.2. Response Characteristics for Hydrogen

Pd is an important medium for detecting hydrogen gas. The Pd electrode acts as a catalyst to selectively absorb hydrogen gas to produce PdH*_x_* within the dielectric layer or to facilitate the reversion process. When hydrogen molecules are adsorbed on the surface of the Pd metal, the hydrogen molecules are decomposed with little or no activation energy barrier into two hydrogen ions to form PdH*_x_* (where *x* < 1). Furthermore, the hydrogen sensor’s inherent selectivity for hydrogen, fast sorption kinetics, and reversibility of hydride formation improve its function.

**Figure 7:**
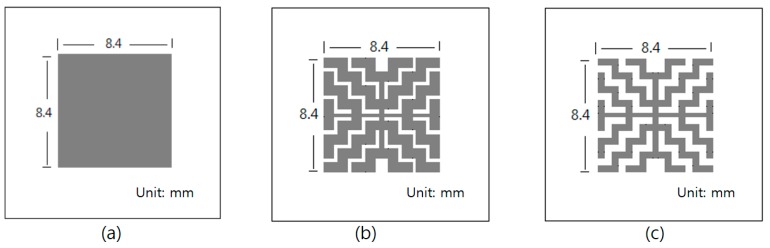
The three types of metal mask patterns used for the Pd electrodes: (**a**) 100%, (**b**) 75%, and (**c**) 50% of Pd mask pattern open.

We fabricated sensors with Pd electrodes consisting of three different patterns in order to assess the effects of Pd electrodes on hydrogen sensors. Figure 7 shows the three types of mask patterns for Pd electrodes, where the gray area is the open area of the mask. The Pd is deposited over the Ta_2_O_5_ layer. The patterns were designed into square or grid shapes, and comprised 100%, 75%, and 50% area ratios of Pd electrodes covered on the Ta_2_O_5_ layer. Figure 7a–c, show the 100% opened mask pattern sized 8.4 × 8.4 mm^2^, 75% opened mask pattern sized 8.4 × 8.4 mm^2^, and 50% opened mask pattern sized 8.4 × 8.4 mm^2^, respectively.

**Figure 8:**
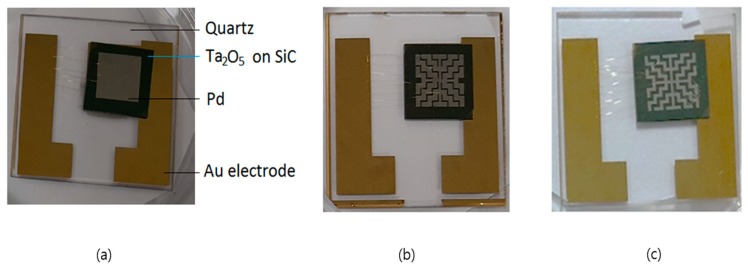
Images of the completed sensors showing the three types of Pd patterns: (**a**) 100%, (**b**) 75%, and (**c**) 50% of Pd mask pattern open.

Figure 8 shows the three types of sensors used in the study attached to quartz plates sized 3 × 3 cm^2^. The sensors on the SiC substrate were interconnected by wire bonding with the electrode pad deposited by gold (Au) on the quartz plate. Figure 8a–c show images of the fabricated sensors with 100%, 75%, and 50% opened masks, respectively.

We examined variations in the capacitance in terms of hydrogen concentration for the three types of sensors with different Pd electrode patterns at temperatures ranging from room temperature to 400 °C. In the MIS-structured sensors, the capacitive-type was less sensitive to temperature changes than the resistive-type [18,19,20] and, thus, temperature correction was no longer required. Before hydrogen was injected, the initial capacitance of the sensors did not vary extensively, regardless of the temperature tested. We first examined the response properties of the sensor when exposed to hydrogen at room temperature. At room temperature, when the concentration of hydrogen increased from zero to 2000 ppm, little variation in capacitance was observed for all three types of sensors.

**Figure 9:**
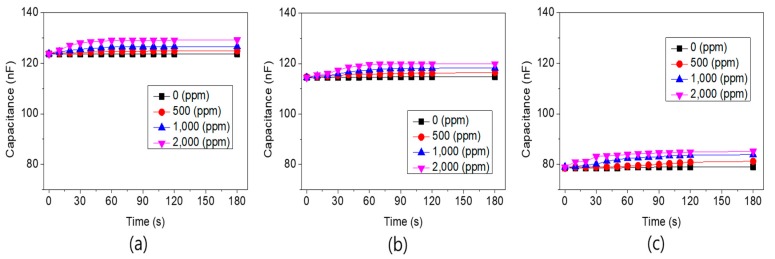
Variability of the capacitance for hydrogen concentration measured at 200 °C, from sensors with (**a**) 100%, (**b**) 75%, and (**c**) 50% area ratios of Pd electrodes on the Ta_2_O_5_ layer.

Figure 9 shows the results obtained at 200 °C. At 200 °C, the capacitance gradually increased when the hydrogen concentration increased to 2000 ppm compared to the capacitance at room temperature. Overall, our results showed that the higher the concentration of hydrogen, the higher the capacitance of the sensor, which was assumed to be due to the increase in PdH_x_ passing through the Pd catalytic electrode as the temperature increased. Figure 9a shows the capacitance of a 100% area ratio of the Pd electrode. The initial value of the capacitance was about 125 nF. Figure 9b,c shows the results obtained from sensors with a 75% and 50% area ratio of the Pd electrode, respectively. The initial values of the capacitance decreased as the area of Pd electrode became smaller, achieving 112 and 80 nF capacitance, respectively.

**Figure 10:**
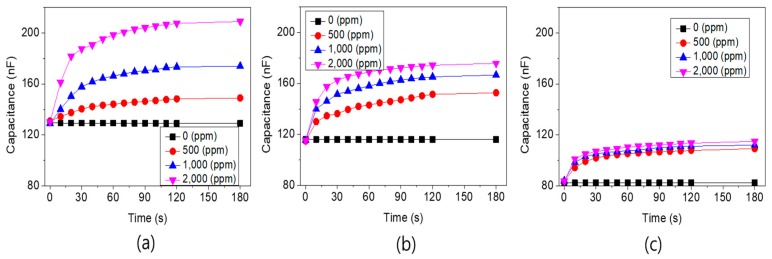
Variability of the capacitance for hydrogen concentration measured at 400 °C, from sensors with (**a**) 100%, (**b**) 75%, and (**c**) 50% area ratio of Pd electrodes on the Ta_2_O_5_ layer.

Figure 10a shows the results of a sensor with a 100% area ratio of the Pd electrode measured at 400 °C. Despite the increase in temperature, the initial capacitance remained at 125 nF with little variation. In the absence of hydrogen, the capacitance remained unchanged at about 125 nF. For hydrogen concentrations of 1000 and 2000 ppm, the capacitance of the sensor rose from 170 nF to 210 nF. The sensitivity was more than 25% per 1000 ppm, when the sensitivity (*S*) was calculated by

(1) $$S=\;\frac{\Delta C}{C_{0}}\;\times 100\;\left(\%\right)$$

where *C*_0_ represents the capacitance in the absence of hydrogen and ΔC represents the change in capacitance when exposed to hydrogen. Overall, the change in capacitance for the increase in hydrogen concentration from zero to 2000 ppm showed almost linear results. Figure 10b shows the results obtained using a sensor with a 75% area ratio of the Pd electrode. For hydrogen concentrations of 1000 and 2000 ppm, the capacitance of the sensor increased from 160 nF to 170 nF. This was less sensitive than the results for a 100% area ratio of the Pd electrode (Figure 10a). Additionally, the increase in rate of capacitance diminished when the hydrogen concentration increased, indicating that the response characteristics to hydrogen concentration were somewhat nonlinear. Finally, Figure 10c shows the results obtained from a sensor with a 50% area ratio of the Pd electrode. For hydrogen concentrations of 1000 and 2000 ppm, the capacitance of the sensor increased from 108 nF to 110 nF. Compared with the previous results shown in Figure 10a,b at 100% and 75% Pd electrode area ratios, the change in capacitance was further reduced, and showed more nonlinear response to the change in hydrogen concentration. These results suggest that the process of changing the adsorbed hydrogen molecules to PdH*_x_* depends strongly on the Pd electrode as a catalyst. Therefore, when sensors respond to hydrogen, our results showed that the higher the area ratio of the Pd electrode, the greater the response properties to hydrogen.

## 4. Conclusions

For sensors to be used at high-temperature environments, thermal stability at high temperatures is required. However, semiconductor devices are sensitive to temperature and, thus, the electrical properties can easily degrade at high temperatures. In this study, the thermal stability at the interface of Ta_2_O_5_/SiC substrate and the dielectric breakdown strength of the Ta_2_O_5_ film formed by RTO at 900 °C or higher were measured. In addition, hydrogen sensors using Ta_2_O_5_ film fabricated on a SiC substrate were studied. For hydrogen sensors, Pd electrodes play an important role as a catalyst. Thus, this study evaluated the role of the Pd electrode in hydrogen sensors with the aim of developing hydrogen sensors that can operate in high-temperature environments. For this purpose, hydrogen sensors using a SiC substrate were fabricated, with the process considering thermal stability. To evaluate the effects of the Pd electrode on the hydrogen sensor, we examined variations in the capacitance according to different hydrogen concentrations for three types of sensors with different Pd electrode patterns as the temperature increased from room temperature to 400 °C. Our results showed that the sensitivity of the sensor increased as the area ratio of the Pd electrode increased from 50% to 100%, and the change in capacitance for the increase in hydrogen concentration showed overall linear results even with some calibration. In addition, when the temperature was raised to 400 °C, the sensitivity of the sensor replaced by variations in capacitance was greatly increased.
